# ﻿A new *Gammarus* species from Xinjiang Uygur Autonomous Region (China) with a key to Xinjiang freshwater gammarids (Crustacea, Amphipoda, Gammaridae)

**DOI:** 10.3897/zookeys.1090.78834

**Published:** 2022-03-25

**Authors:** Kui Zhang, Jun Wang, Yihao Ge, Jishun Ma, Qiong Zhou

**Affiliations:** 1 Key Laboratory of Freshwater Animal Breeding, Ministry of Agriculture and Rural Affair/ Engineering Research Center of Green development for Conventional Aquatic Biological Industry in the Yangtze River Economic Belt, Ministry of Education, College of Fisheries, Huazhong Agricultural University, Wuhan 430070, China Huazhong Agricultural University Wuhan China; 2 Key Laboratory of Ecological Impacts of Hydraulic-Projects and Restoration of Aquatic Ecosystem of Ministry of Water Resources, Institute of Hydroecology, Ministry of Water Resources and Chinese Academy of Sciences, Wuhan 430079, China Institute of Hydroecology, Ministry of Water Resources and Chinese Academy of Sciences Wuhan China

**Keywords:** Amphipoda diversity, mitochondrial DNA, morphology, new species, nuclear DNA, taxonomy, Xinjiang

## Abstract

A new species of the genus *Gammarus* Fabricius, 1775 is described and illustrated from Xinjiang Uygur Autonomous Region, China. *Gammaruszhouqiongi***sp. nov.** is characterized by pereopods III–IV with long straight setae on posterior margins; inner ramus of uropod III more than twice as long as peduncle, reaching 0.7 times the length of outer ramus; inner ramus with plumose setae, and outer ramus with both plumose setae and long simple setae. Detailed morphological comparisons with related species are discussed. The K2P distances for each marker (CO1, 16S, 28S, and EF1α) of the new species differ from those of other *Gammarus* species in Xinjiang. Both phylogenetic trees based on separate (CO1, 16S, 28S, and EF1α) and combined (CO1+16S+28S+EF1α) markers show that the new species is an independent branch. A key to identify *Gammarus* species in Xinjiang is provided.

## ﻿Introduction

The genus *Gammarus* Fabricius, 1775 is distributed in Eurasia and North America, and is one of the genera with the highest species richness in freshwater amphipods (Zhao et al. 2017). Previous studies suggest that *Gammarus* originated in the Tethys Ocean, and the regression of Paratethys played an important role in its dispersal to Eurasia (Hou et al. 2011). The Xinjiang Uygur Autonomous Region (Xinjiang afterwards) is located between the Lake Baikal and the Ponto-Caspian Basin, and is one of the most major zones of endemic amphipod species diversity (Väinölä et al. 2008), acting as a crossroad among the various regions of the Palaearctic Realm. However, only eight *Gammarus* species are described in Xinjiang. Particularly, seven of them are endemic species, including *Gammarustastiensis* Hou, *G.decorosus* Meng, Hou & Li, *G.brevipodus* Hou & Li, *G.takesensis* Hou & Li, *G.tianshan* Zhao, Meng & Hou, *G.simplex* Zhao, Meng & Hou, *G.liuruiyui* Zheng, Hou & Li (Hou 2002; Meng et al. 2003; Hou et al. 2004; Zhao et al. 2017; Zheng et al. 2020) and one is a widespread species (*G.lacustris* Sars, 1863) in alpine lakes. The amphipod diversity of Xinjiang still remains incompletely understood.

**Figure 1.**
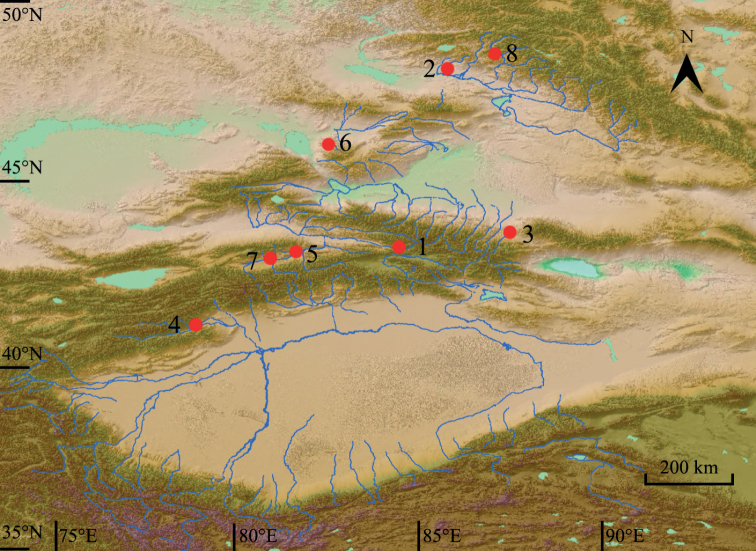
Distribution map of *Gammarus* species from Xinjiang (China). Type localities are shown for the species **1–8**. **1***Gammarusbrevipodus* Hou & Li, 2004 **2***G.zhouqiongi* sp. nov. **3***G.decorosus* Meng, Hou & Li, 2003 **4***G.liuruiyui* Zheng, Hou & Li, 2020 **5***G.takesensis* Hou & Li, 2004 **6***G.tastiensis* Hou, 2002 **7***G.tianshan* Zhao, Meng & Hou, 2017 **8***G.simplex* Zhao, Meng & Hou, 2017 (map data from GEBCO Compilation Group [2020]).

During our field surveys in Xinjiang between 2012–2020, a new species was discovered based on morphological and molecular analyses. To further identify and understand the evolutionary origins of the new species, phylogenetic analyses of *Gammarus* in Xinjiang were performed. The distributions of endemic species of the genus *Gammarus* in Xinjiang are presented in Fig. 1.

## ﻿Materials and methods

### ﻿Sampling

Specimens were collected from the streams and adjacent puddles with fine-meshed hand nets (500 μm). Samples were stored in 95% ethanol in the field, and then deposited at -80 °C for long-term preservation. Type specimens are lodged in the College of Fisheries, Huazhong Agricultural University, Wuhan (China).

### ﻿Morphometrics

All dissected appendages were examined and drawn using a Leica DM2500 compound microscope equipped with a drawing tube. The body length was measured from the base of the first antenna to the end of the telson while the specimens were kept straight. Terminology and taxonomic description referred to Zhao et al. (2017). Nomenclature of the setae of mandibular palps followed Cole (1980).

### ﻿DNA sequencing and phylogenetic analyses

We did not obtain samples of *G.simplex* during field surveys, and no relevant record was accessible in GenBank. Genomic DNA was extracted using the Animal Genomic DNA Kit (Tsingke Biotech, Beijing). K2P distances based on each marker were calculated in MEGA 6 (Tamura et al. 2013). We utilized two mitochondrial and two nuclear markers, previously used for *Gammarus* phylogeny (Hou et al. 2007, 2011, Copilas-Ciocianu et al. 2019), based on both separate and combined markers to understand the phylogenetic relationships between *G.zhouqiongi* sp. nov. and other *Gammarus* species in Xinjiang. The mitochondrial markers included the fragments for cytochrome c oxidase 1 (CO1) and 16S ribosomal RNA (16S), whereas the nuclear markers included the fragments for 28S ribosomal RNA (28S) and elongation factor 1-alpha (EF1α). The primers are presented in Table 1. Raw sequences were aligned with muscle (Edgar 2004) and translated to amino acids to check for potential pseudogenes in MEGA 6. We selected *Jesogammarusdebilis* Hou & Li, 2005, *Jesogammarushebeiensis* Hou & Li, 2004 and *Rhipidogammarusrhipidiophorus* Catta, 1878 for EF1α as the outgroup. The details of newly obtained sequences in this study and the sequences downloaded from GenBank are shown in Table 2.

**Table 1.** Primer sequences of PCR products for target genes.

Gene	Primer	Sequence (5'–3')	Reference
CO1	LCO1490	GGTCAACAAATCATAAAGATATTGG	Folmer et al. (1994)
	HCO2198	TAAACTTCAGGGTGACCAAAAAAT	Folmer et al. (1994)
	LCO3	TCNACHAAYCATAAAGAYATTGGTAC	Krebes et al. (2010)
16S	16STf	GGTAWHYTRACYGTGCTAAG	MacDonald et al. (2005)
	16Sbr	CCGGTTTGAACTCAGATCATGT	Palumbi et al. (1991)
28S	28F	TTAGTAGGGGCGACCGAACAGGGAT	Hou et al. (2007)
	28R	GTCTTTCGCCCCTATGCCCAACTGA	Hou et al. (2007)
EF1α	EF1αF	CACTACTGGTCATCTCATCTAC	Hou et al. (2011)
	EF1αR	ACTTCCAGGAGAGTCTCAAAC	Hou et al. (2011)

We selected the best-fit models by Akaike information criterion (AICc) in PartitionFinder (Lanfear et al. 2012). For phylogenetic analysis, we utilized the IQ-Tree 1.4.2 (Nguyen et al. 2015) to construct a phylogenetic tree based on the maximum likelihood (ML) method. 1000 bootstrap replicates were performed to assess nodal support.

**Table 2.** Taxon information and Genbank numbers for the complete dataset.

Taxon	Coordinates	CO1	16S	28S	EF1α	Reference
*Gammarusbrevipodus*	43.28N, 84.28E	MW723045	MW729654	MW729697	MW749858	This study
*G.zhouqiongi*1	46.76N, 84.42E	MW723044	MW729651	MW729694	MW749855	This study
*G.zhouqiongi*2	48.08N, 86.35E		MW729649	MW729692	MW749853	This study
*G.decorosus*	43.80N, 87.60E	JF965875		JF965684	JF966031	Hou et al. 2011
*G.lacustris*	47.24N, 88.47E	MW717900	MW729628	MW729674	MW749832	This study
*G.liuruiyui*	40.88N, 78.19E	MK455899		MK455898		Zheng et al. 2020
*G.takesensis*	43.63N ,81.80E	MW723041	MW729638	MW729681	MW749842	This study
*G.tastiensis*	45.95N, 82.57E	MW723046	MW729655	MW729698	MW749859	This study
*G.tianshan*	43.1N, 81.1E	EF570327	EF582873	EF582971		Hou et al. 2007
*Jesogammarusdebilis*	39.5N, 115.8E	EF570351	EF582846	EF582997		Hou et al. 2007
*J.hebeiensis*	40.4N, 115.9E	EF570352	EF582847	EF582998		Hou et al. 2007
*Rhipidogammarusrhipidiophorus*	40.28N, 9.63E				JF966114	Hou et al. 2011

## ﻿Results

### ﻿Molecular analyses

**Figure 2.**
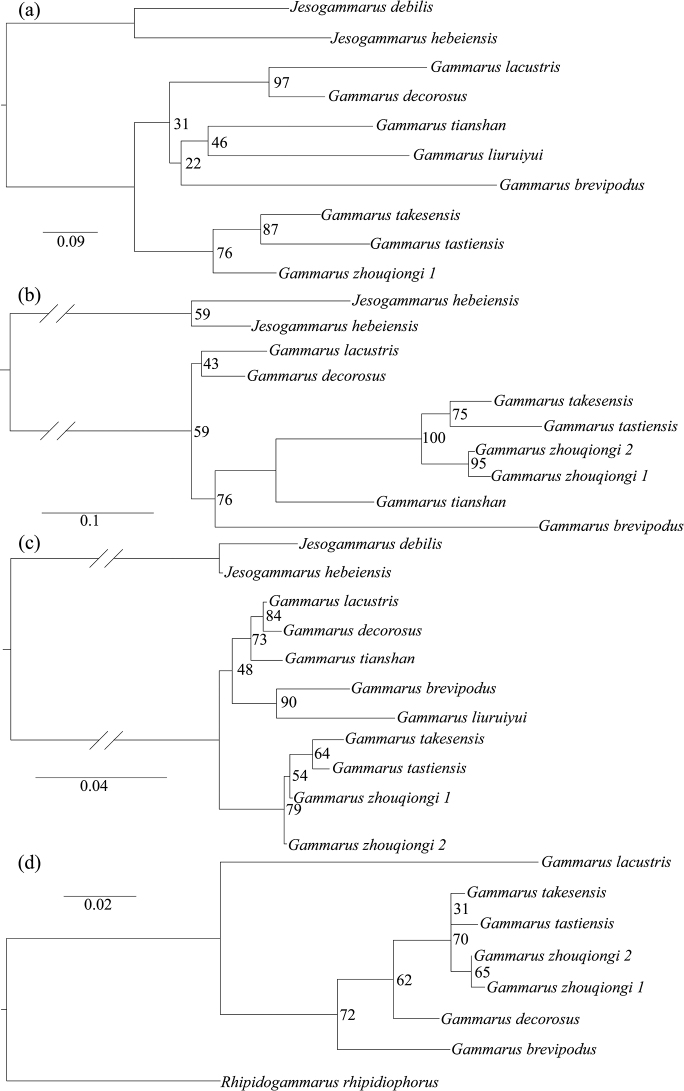
Maximum likelihood trees for *Gammarus* from Xinjiang based on the four separate markers: **a**CO1**b**16S**c**28S**d**EF1α. Numbers near the nodes are bootstrap values.

**Figure 3.**
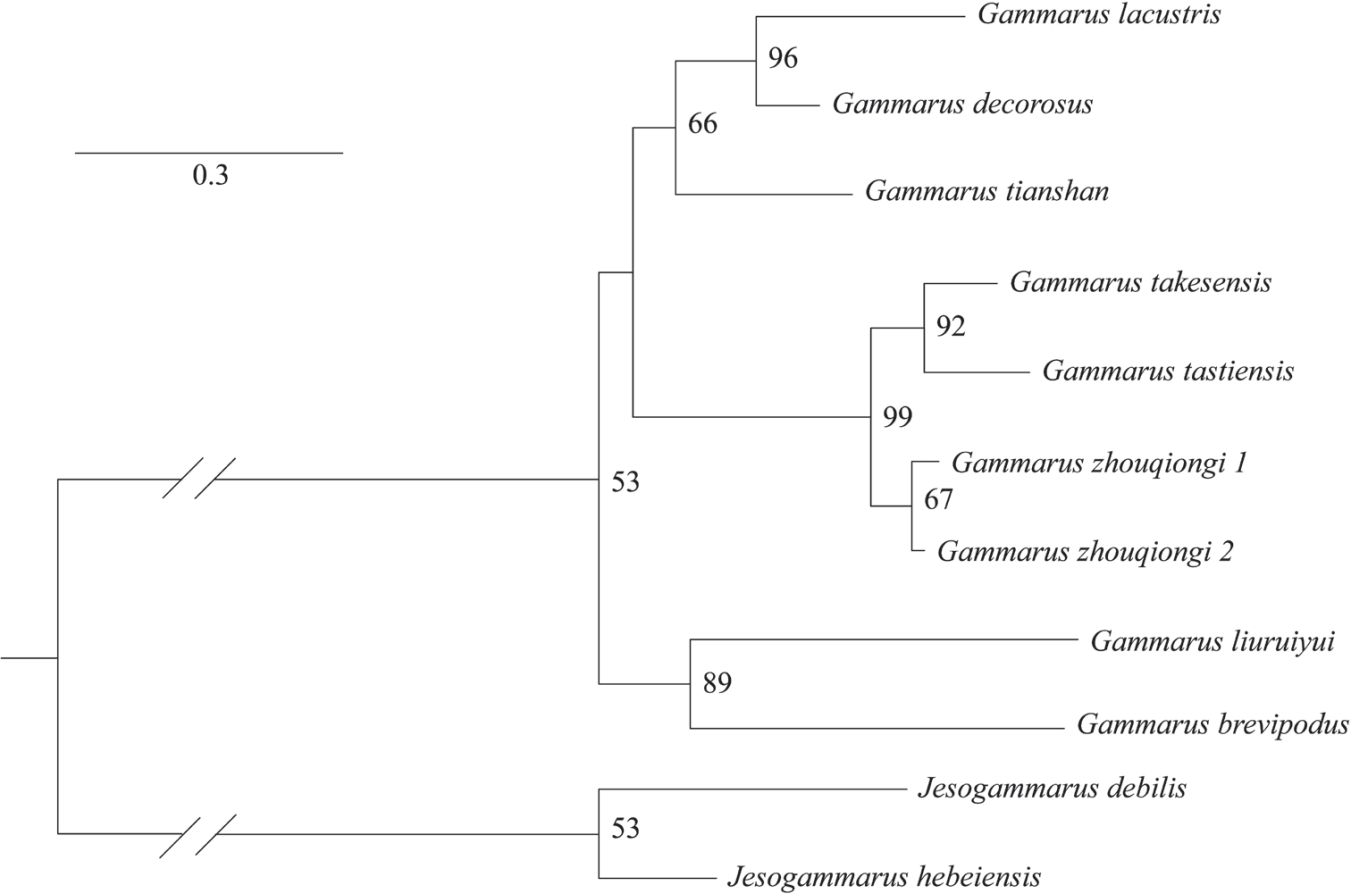
Maximum likelihood tree for *Gammarus* from Xinjiang based on combined markers (CO1+16S+28S+EF1α). Numbers near the nodes are bootstrap values.

The values of K2P distances between *Gammaruszhouqiongi* sp. nov. and other *Gammarus* species in Xinjiang (*G.simplex* excluded) ranged between 16.6%–32.4% for CO1, 11.0%–39.3% for 16S, 1.2%–6.3% for 28S and 1.3%–9.6% for EF1α (Table 3), respectively. In contrast, many studies relevant to *Gammarus* reported similar or lower levels of divergence. Hou et al. (2014) showed 11.2–20.3% for CO1 and 1.1–3.7% for 28S (uncorrected p-distance), respectively, among *Gammarus* species in Luliang Mts and Taihang Mts. Copilas-Ciocianu et al. (2019) found 13.3% for CO1, 4.3% for 16S, 0.4% for 28S and 1.8% for EF1α, respectively, between *G.hamaticornis* and *G.kischineffensis*. The genetic clusters of *Gammaruszhouqiongi* sp. nov. were clearly distinguished from other species (Figs 2, 3), suggesting one new species to science.

**Table 3.** Kimura 2-parameter pairwise genetic distances of *Gammarus* in Xinjiang.

		Species	1	2	3	4	5	6	7	8
CO1 (below diagonal)/16S (above diagonal)	1	*Gammarusbrevipodus*		0.393	0.281	0.321		0.336	0.329	0.343
	2	*G.zhouqiongi*1	0.324		0.248	0.239		0.110	0.144	0.210
	3	*G.decorosus*	0.349	0.262		0.085		0.232	0.258	0.170
	4	*G.lacustris*	0.389	0.297	0.215			0.231	0.282	0.196
	5	*G.liuruiyui*	0.316	0.308	0.265	0.329				
	6	*G.takesensis*	0.347	0.166	0.267	0.324	0.326		0.104	0.193
	7	*G.tastiensis*	0.322	0.190	0.264	0.352	0.355	0.177		0.229
	8	*G.tianshan*	0.359	0.288	0.301	0.327	0.316	0.313	0.282	
28S (below diagonal)/EF1α (above diagonal)	1	*G.brevipodus*		0.067	0.053	0.132		0.065	0.069	0.061
	2	*G.zhouqiongi*1	0.053		0.029	0.096		0.013	0.017	0.044
	3	*G.decorosus*	0.044	0.037		0.098		0.031	0.031	0.022
	4	*G.lacustris*	0.040	0.033	0.007			0.112	0.118	0.120
	5	*G.liuruiyui*	0.053	0.063	0.052	0.051				
	6	*G.takesensis*	0.058	0.017	0.042	0.038	0.071		0.011	0.039
	7	*G.tastiensis*	0.049	0.012	0.039	0.033	0.066	0.014		0.042
	8	*G.tianshan*	0.044	0.039	0.017	0.014	0.054	0.045	0.037	

## ﻿Taxonomy

### ﻿Family Gammaridae Leach, 1814

#### ﻿Genus Gammarus

Taxon classificationAnimaliaAmphipodaGammaridae

Fabricius, 1775

8C5DDD5E-5CFD-544B-A5FE-E0F5D2DB1D29

##### Type species.

*Gammaruspulex* (Linnaeus, 1758).

#### ﻿ Gammarus zhouqiongi sp. nov.

Taxon classificationAnimaliaAmphipodaGammaridae

8DD5FE36-1847-5F25-A704-4575574D15FD

http://zoobank.org/0120F1C0-D50B-45C7-A9C0-B32650AAD6F2

**Figure 4.**
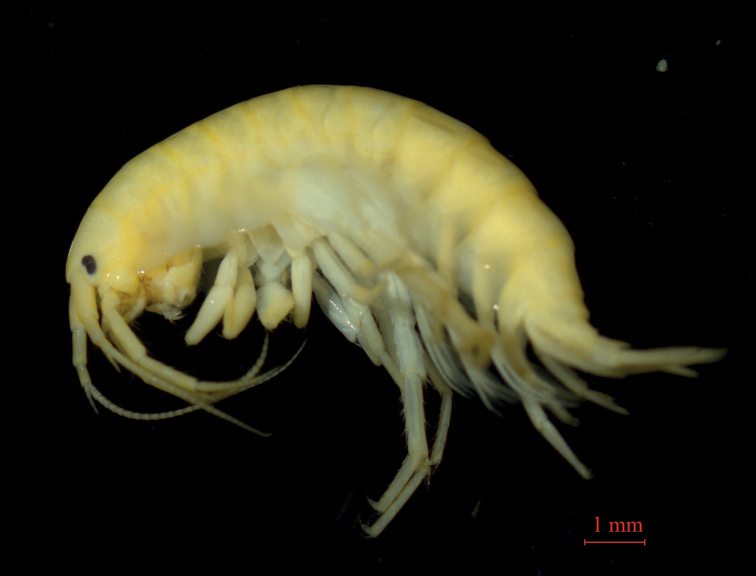
*Gammaruszhouqiongi* sp. nov., holotype.

**Figure 5.**
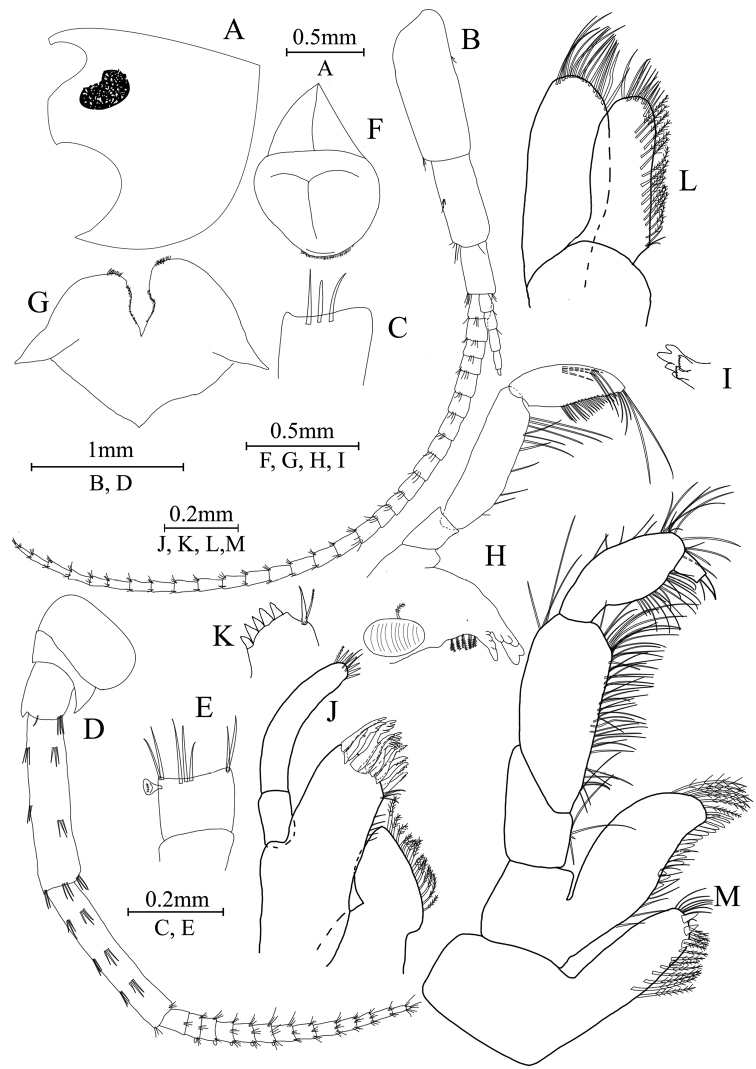
*Gammaruszhouqiongi* sp. nov., male holotype **A** head **B** antenna I **C** flagellar article of antenna I with aesthetasc **D** antenna II **E** calceoli of antenna II **F** upper lip **G** lower lip **H** left mandible **I** incisor and lacinia mobilis of right mandible **J** left maxilla I **K** distal part of palp article II of right maxilla I **L** maxilla II **M** maxilliped.

**Figure 6.**
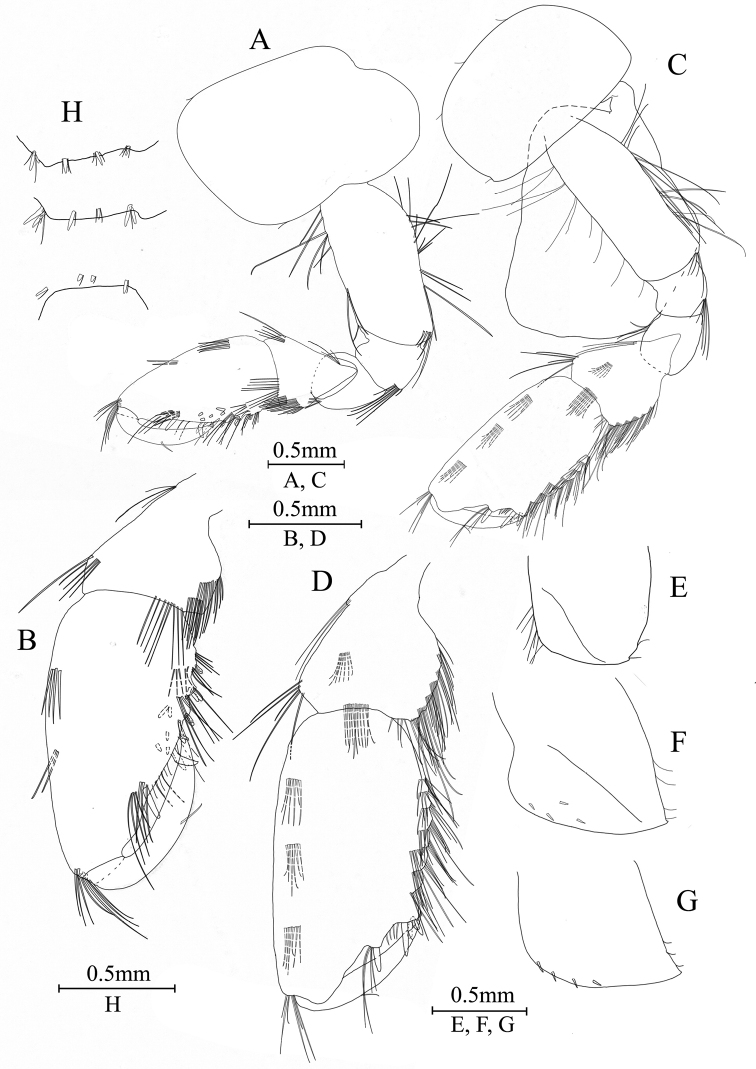
*Gammaruszhouqiongi* sp. nov., male holotype **A** gnathopod I **B** propodus and dactylus of gnathopod I **C** gnathopod II **D** propodus and dactylus of gnathopod II **E** epimeral plate I **F** epimeral plate II **G** epimeral plate III **H** dorsal margins of urosomites I–III.

**Figure 7.**
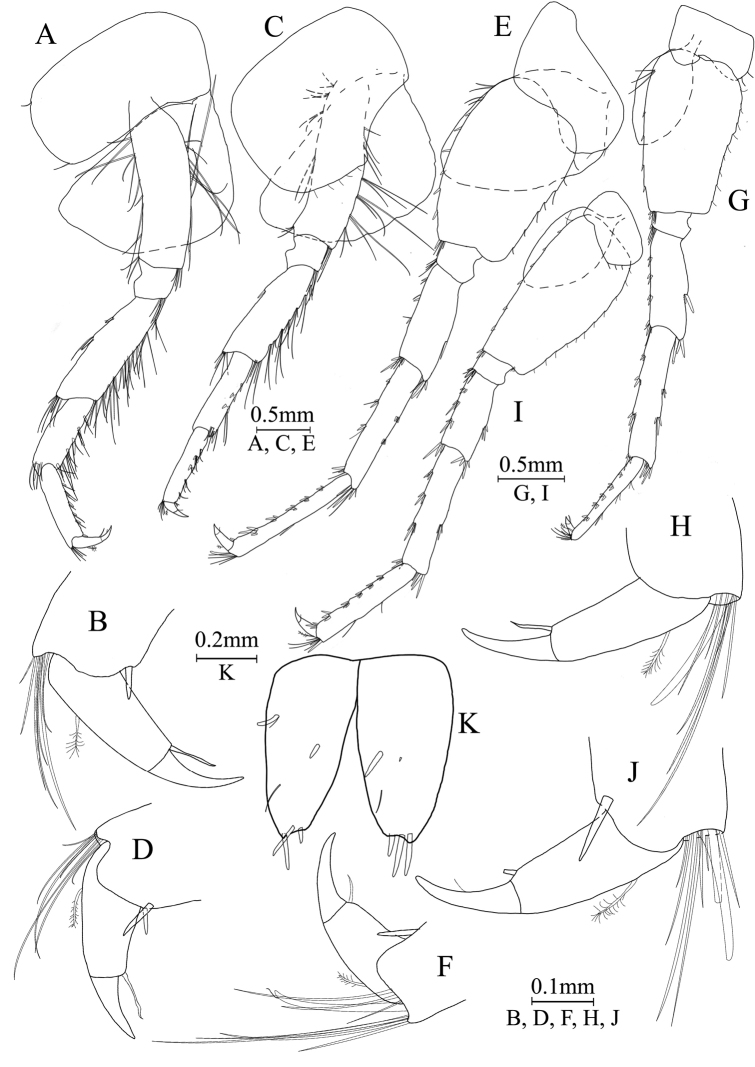
*Gammaruszhouqiongi* sp. nov., male holotype **A** pereopod III **B** dactylus of pereopod III **C** pereopod IV **D** dactylus of pereopod IV **E** pereopod V **F** dactylus of pereopod V **G** pereopod VI **H** dactylus of pereopod VI **I** pereopod VII **J** dactylus of pereopod VII **K** telson.

**Figure 8.**
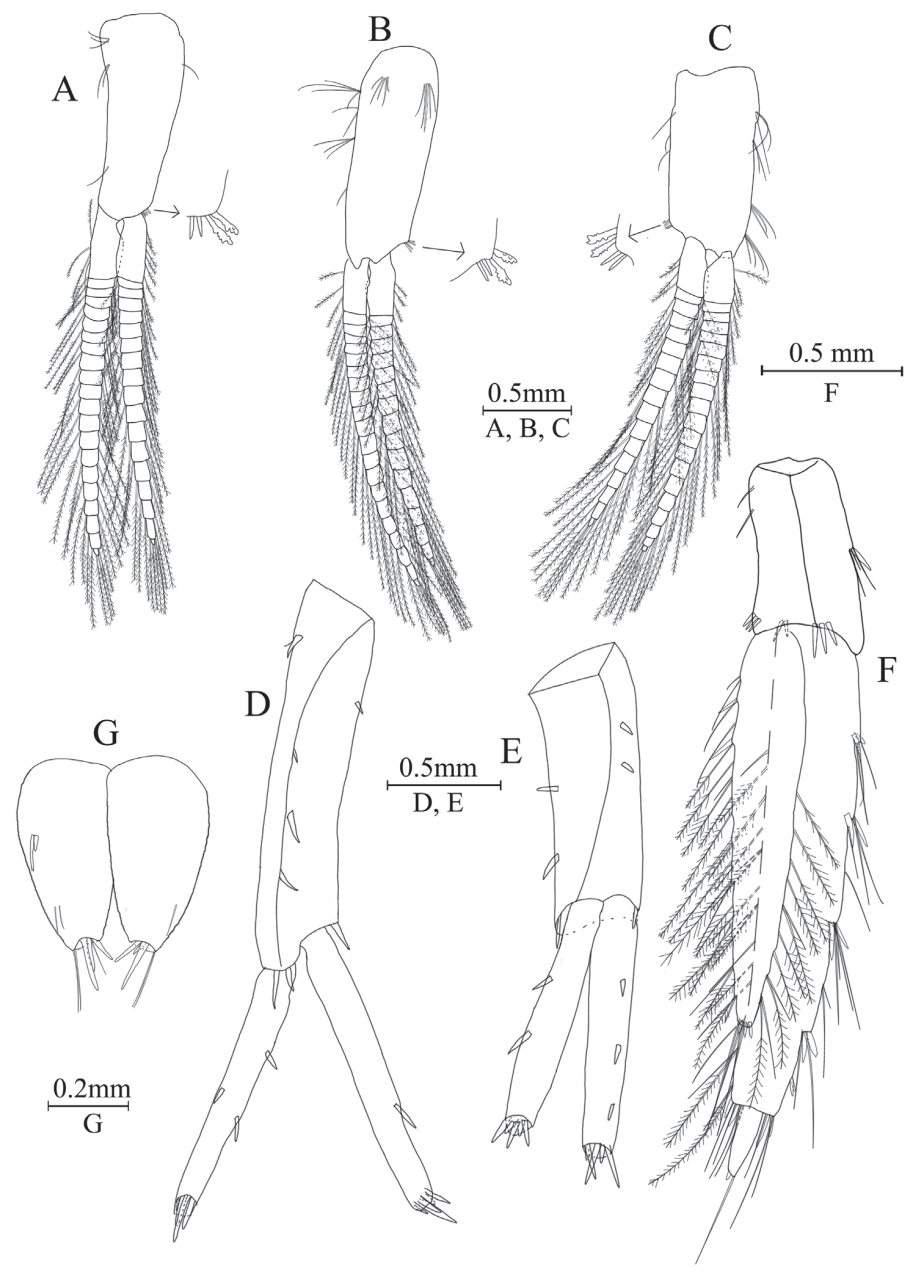
*Gammaruszhouqiongi* sp. nov. **A–F** male holotype **G** female paratype **A** plepod I **B** pleopod II **C** pleopod III **D** uropod I **E** uropod II **F** uropod III **G** telson.

**Figure 9.**
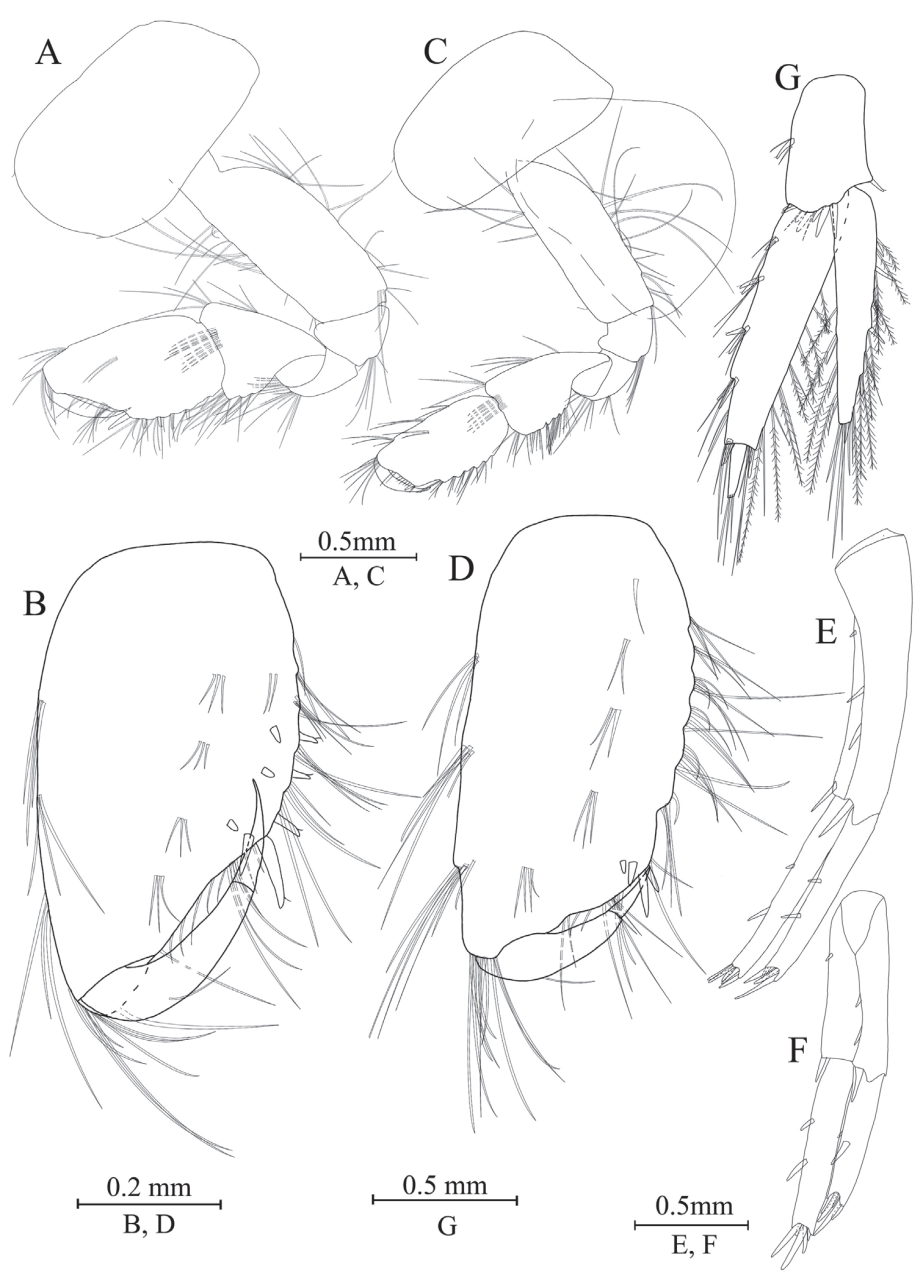
*Gammaruszhouqiongi* sp. nov., female paratype (GAHBH-002) **A** gnathopod I **B** propodus of gnathopod I **C** gnathopod II **D** propodus of gnathopod II **E** uropod I **F** uropod II **G** uropod III.

**Figure 10.**
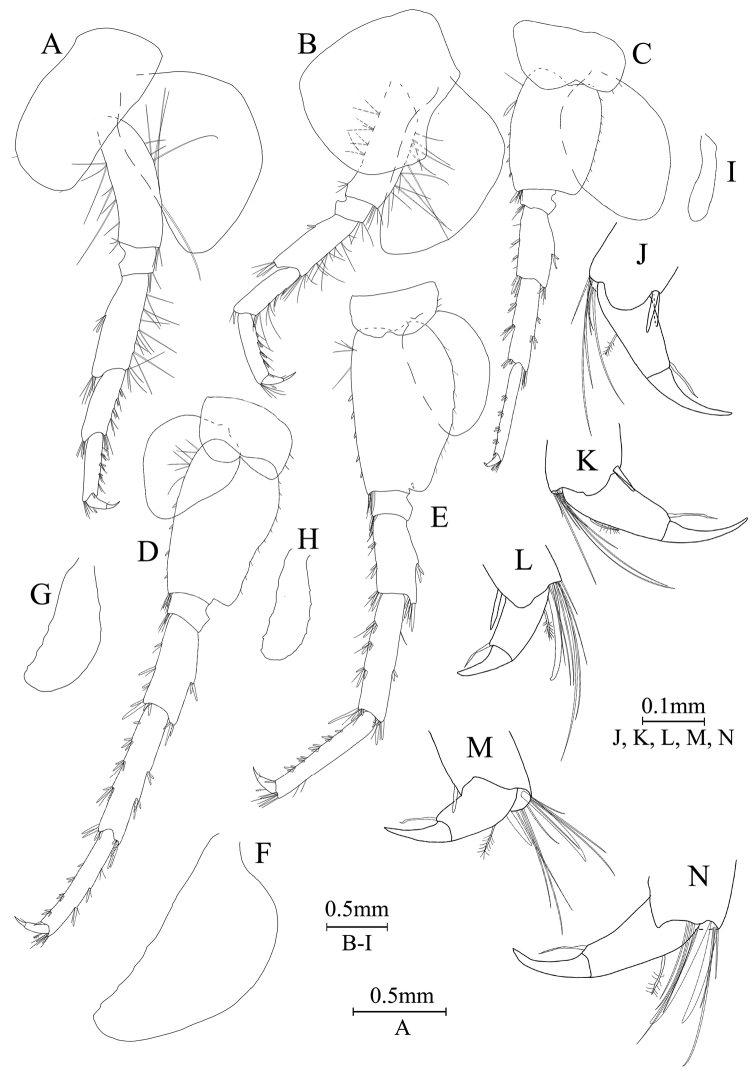
*Gammaruszhouqiongi* sp. nov., female paratype (GAHBH-002) **A** pereopod III **B** pereopod IV **C** pereopod V **D** pereopod VI **E** pereopod VII **F** oostegite of gnathopod II **G** oostegite of pereopod III **H** oostegite of pereopod IV **I** oostegite of pereopod V **J** dactylus of pereopod III **K** dactylus of pereopod IV **L** dactylus of pereopod V **M** dactylus of pereopod VI **N** dactylus of pereopod VII.

Figs 4 , 5 , 6 , 7 , 8 , 9 , 10

##### Material examined.

***Holotype***: male (GAHBH-001), 14.9 mm, Habahe County (48.08°N, 86.35°E), altitude 528 m, Xinjiang Uygur Autonomous Region, China, October 16, 2020, collected by Kui Zhang. ***Paratypes***: female (GAHBH-002), 12.3 mm; five males and three females (GAHBH003-010), same data as holotype. three males and two females (GAKLY001-005), Emin County (46.76°N, 84.42°E), altitude 991 m, Xinjiang Uygur Autonomous Region, China, July 12, 2015, collected by Jun Wang and Yihao Ge.

##### Etymology.

The specific name was to thank Professor Zhou for funding this study.

##### Diagnosis.

Peduncle articles IV–V of antenna II with clusters of short setae; merus to carpus of pereopod III with clusters of long setae that exceed the width of the underlying segment on posterior margins; epimeral plates III with subacute posterodistal corners; inner ramus of uropod III more than twice times as long as peduncle, reaching 0.7 times the length of outer ramus, both inner and outer margins of inner ramus and the inner margins of outer ramus with plumose setae, and outer margin of outer ramus with long simple setae.

##### Description of male holotype.

(GAHBH-001), 14.9 mm.

***Head*.** (Fig. 5A): eyes reniform, inferior antennal sinus deep.

Antenna I (Fig. 5B, C): peduncle articles I–III in length ratio 1.0: 0.7: 0.4 bearing short setae; flagellum with 30 articles, most with aesthetascs; accessory flagellum with five articles; both primary and accessory flagella bearing small setae distally.

Antenna II (Fig. 5D, E): peduncle articles III–V in length ratio 1.0: 3.0: 2.9, peduncle article III with lateral setae, articles IV and V of peduncle with clusters of lateral and medial setae; flagellum with 14 articles, each article with setae along ventral margins; articles II–VI with calceoli.

Upper lip (Fig. 5F): ventral margin rounded, with minute setae on the distal part.

Mandible (Fig. 5H, I): left mandible incisor with five teeth; lacinia mobilis with four teeth; spine row with five pairs of plumose setae; articles I–III of palp in length radio 1.0: 2.3: 3.0, second article of palp with 11 marginal setae, article III with three A-setae, three B-setae, 19 D-setae, and five E-setae apically; incisor of right mandible with four teeth; lacinia mobilis bifurcate, with a row of small teeth at the end.

Lower lip (Fig. 5G): inner lobes lacking, outer lobes covered with thin setae.

Maxilla I (Fig. 5J, K): asymmetrical, left inner plate with 14 plumose setae on medial margin; outer plate with 11 robust serrated apical spines, each spine with small teeth; second article of left palp with six slender spines, two long setae and one spine with small setae; second article of right palp with five stout spines, one stiff seta and one slender spine.

Maxilla II (Fig. 4L): inner plate with 15 plumose facial setae in an oblique row; inner and outer plates with long setae apically.

Maxilliped (Fig. 4M): inner plate with three stout apical spines, one subapical spine, eight simple setae, and 12 plumose setae; outer plate bearing a row of blade spines and six plumose setae apically; article IV of palp hooked, with a group of setae at hinge of unguis.

***Pereon*.** Gnathopod I (Fig. 6A, B): coxal plate bearing one seta on both anterior and posterior margins; basis with long setae on anterior and posterior margins; carpus 1.1 times as long as wide, 0.7 times as long as propodus; propodus oval, palm with one medial spine and 16 spines on posterior margin and surface; dactylus with one seta on outer margin.

Gnathopod II (Fig. 6C, D): coxal plate bearing three setae and one seta on anterior and posterior margins; basis with long setae on anterior and posterior margins; carpus 1.2 times as long as wide, 0.6 times as long as propodus; propodus subrectangular, palm margin with one medial spine and four spines on lateral posterior margin and surface; dactylus with one seta on outer margin.

Pereopod III (Fig. 7A, B): both anterior and posterior margins of coxal plate bearing one setae; basis elongate, with setae along anterior and posterior margins; merus with two spines accompanied by one seta on anterior margin and clusters of long setae on posterior margin, 1 spine accompanied by setae in anterodistal corner; carpus with five spines accompanied by setae on posterior margin, one spine with setae in anterodistal corner; propodus with five spines accompanied by setae on posterior margin and one spine on posterodistal corner; dactylus with one plumose seta on anterior margin, and one setae at hinge of unguis.

Pereopod IV (Fig. 7C, D): coxal plate concave, bearing five setae on posterior margin; basis with clusters of setae on anterior and posterior margin; merus has several clusters of setae on posterior margin and 1 spine on anterior margin, anterodistal corner with one spine accompanied by setae; carpus with five spines on posterior margin and two spines accompanied by setae on posterodistal corner; propodus with seven spines accompanied by setae on posterior margin and two spines on posterodistal corner; dactylus with one plumose seta on anterior margin and one seta at hinge of unguis.

Pereopod V (Fig. 7E, F): coxal plate bearing two setae on posterior margin; basis expanded, with setae and six spines on anterior margin, anterodistal corner with one spine and three setae, posterior margin with seven setae; merus with three spines accompanied by setae on both anterior margin and anterodistal corner, posterior margin with one spine and posterodistal corner with three spines; carpus with three or two groups of spines on anterior margin and posterior margin, respectively; propodus with five groups of spines on anterior margin; dactylus with one plumose seta on posterior margin, and one seta at hinge of unguis.

Pereopod VI (Fig. 7G, H): coxal plate bearing two setae on posterior margin; basis expanded, with three setae and four spines on anterior margin, anterodistal corner with two spines accompanied by setae, posterior margin with nine setae; merus with three pairs of spines on anterior margin and three spines accompanied by setae on anterodistal corner, posterior margin with one pair of spines and posterodistal corner with three spines; carpus with three or two groups of spines on anterior margin and posterior margin, respectively; propodus with five groups of spines on anterior margin, posterior margin with one spine and five setae; dactylus with one plumose seta on posterior margin, and one seta at hinge of unguis.

Pereopod VII (Fig. 7I, J): coxal plate bearing three setae on posterior margin; basis expanded, with two setae and six spines on anterior margin, anterodistal corner with three spines, eleven setae on posterior margin and one spines accompanied by three setae on posterodistal corner, respectively; both mersus and carpus with three spines on anterior margin and one spine on posterior margin; propodus with five groups of spines on anterior margin and two setae on posterior margin; dactylus with one plumose seta on posterior margin and one seta at hinge of unguis.

Coxal gills (Figs 6C, 7A–E): coxal gill of gnathopod II longer than basis; gills of pereopod III–V are almost as long as their basis; gills of pereopod VI–VII are shorter than their basis.

***Pleon*.** Epimeral plates (Fig. 6E–G): plate I ventrally rounded, bearing seven setae on anteroventral margin and two setae on posterior margin; plate II with four spines on ventral margin and four setae on posterior margin, posterodistal corner blunt; plate III with four spines on ventral margin and three setae on posterior margin, posterodistal corner subacute.

Pleopods (Fig. 7A–C): similar, peduncle with two retinacula accompanied by two or three setae; outer ramus slightly shorter than inner ramus, both inner and outer rami fringed with plumose setae.

***Urosome*.** Urosomites (Fig. 6H): urosomite I with two-one-one-two spines accompanied by setae on dorsal margin; urosomite II with two-one-one-two spines accompanied by setae on dorsal margin; urosomite III with one-one-one-one spine accompanied by one seta.

Uropods I–III (Fig. 8D–F): uropod I peduncle with one basofacial spine, one and three spines on inner and outer margins, with one and two spines on inner and outer distal corners, respectively; inner ramus with one spine on inner margin; outer ramus with one and two spines on inner and outer margins, respectively; both rami with five terminal spines. Uropod II peduncle with two spines on both inner and outer margins and one distal spine on each corner; inner ramus with three spines on inner margin, outer ramus with two spines on outer margin, both rami with five terminal spines. Uropod III peduncle with one spine accompanied by three setae and eight distal spines; inner ramus about 2.4 times as long as peduncle, reaching 0.7 times the length of outer ramus, with two spines on inner margin, both inner margin and outer margin have plumose setae; proximal article of outer ramus with five pairs of spines accompanied by several simple setae on outer margin, inner margin with both simple setae and plumose setae, and four distal spines accompanied by long simple setae; terminal article with long simple setae.

Telson (Fig. 7K): deeply cleft, approximately as long as wide; left lobe with two spines and two setae on surface; right lobe with one spine and one single seta; each lobe bearing three distal spines.

##### Description of paratype female.

(GAHBH-002). 12.3 mm

***Pereon***. Gnathopod I (Fig. 9A, B): coxal plate bearing one seta on both anterior and posterior margins; basis with long setae on anterior and posterior margins; propodus oval, palm with 8 spines on posterior margin and surface; dactylus with one seta on outer margin.

Gnathopod II (Fig. 9C, D): coxal plate bearing three setae and one seta on anterior and posterior margins; basis with long setae on anterior and posterior margins; propodus subrectangular, palm margin with four spines on lateral posterior margin and surface; dactylus with one seta on outer margin.

Pereopods III–VII (Fig. 10A–E, J–N): similar to those of males.

Oostegite (Fig. 9F–I): oostegite of gnathopod II broad, oostegites of pereopods III–V elongated and oostegite of pereopod V smallest.

***Urosome***. Uropods I–III (Fig. 9G–F): uropod I peduncle with one or three spines on inner and outer margins respectively, with one spine on both inner and outer distal corners; both rami with two spines on inner margin and five terminal spines. Uropod II peduncle with one or two spines on inner and outer margins respectively and one distal spine on each corner; both rami with two spines on inner margin and five terminal spines. Uropod III peduncle with one spine accompanied by setae and eight distal spines; inner ramus about 2 times as long as peduncle, reaching 0.8 times the length of outer ramus, with four spines on inner margin and one distal spine accompanied by long setae, both inner and outer margins have plumose setae; proximal article of outer ramus with one spine and three pairs of spines accompanied by several simple setae on outer margin, inner margin with both simple setae and plumose setae, and four distal spines accompanied by long simple setae; terminal article with long simple setae.

Telson (Fig. 8G): deeply cleft, approximately as long as wide; left lobe with two spines and two setae on surface; right lobe with two setae; each lobe bearing three distal spines.

##### Habitat.

This species was collected from streams and the adjacent small puddles, usually under big rocks.

##### Remarks.

The new species *Gammaruszhouqiongi* sp. nov. is similar to *G.takesensis* in pereopods III and IV with straight setae on posterior margin; epimeral plates III with subacute posterodistal corners; and inner ramus of uropod III about 0.7 times as long as outer ramus. It differs from *G.takesensis* (*G.takesensis* in parentheses) by accessory flagellum of antenna I with five articles (four articles); inner and outer margins of inner ramus and the inner margins of outer ramus of uropod III with long plumose setae (short plumose setae); posterodistal corner of basis of pereopod VII with spines and setae (only with setae).

*Gammaruszhouqiongi* sp. nov. is also similar to *G.tastiensis* in peduncle articles IV–V of antenna II with short setae; pereopods III and IV with long and straight setae on posterior margin; both inner and outer margins of inner ramus and the inner margins of outer ramus of uropod III with plumose setae, and outer margin of outer ramus of uropod III with simple setae. It can be distinguished from *G.tastiensis* by the following characters (*G.tastiensis* in parentheses): inner ramus of uropod III more than 2 times as long as peduncle (inner ramus uropod III less than 2 times as long as peduncle); pereopods III–V are slender (strong).

A comparison between *Gammarus* species in Xinjiang is presented in the following key.

### ﻿Key to the *Gammarus* species from Xinjiang Uygur Autonomous Region (China)

**Table:** 

1	Eyes present	**2**
–	Eyes absent	***Gammarusliuruiyui***
2	Uropod III inner ramus less than 0.6 times the length of outer ramus	**3**
–	Uropod III inner ramus more than 0.6 times the length of outer ramus	**5**
3	Pereopod III–IV posterior margins and uropod III bearing sparse setae	***G.brevipodus***
–	Pereopod III–IV posterior margins and uropod III bearing normally distributed setae	**4**
4	Peduncle articles IV–V of antenna II with long setae and epimeral plate III with blunt posterodistal corner	***G.simplex***
–	Peduncle articles IV–V of antenna II with short setae and epimeral plate III with subacute posterodistal corner	***G.tianshan***
5	Uropod III outer ramus with plumose setae	**6**
–	Uropod III outer ramus with simple setae	**7**
6	Telson bearing short setae and epimeral plate III with acute posterodistal corner	***G.lacustris***
–	Telson bearing long setae and epimeral plate III with blunt posterodistal corner	***G.decorosus***
7	Posterodistal corner of basis of pereopod VII with setae	***G.takesensis***
–	Posterodistal corner of basis of pereopod VII with spines	**8**
8	Pereopod V–VII are slender and inner ramus uropod III more than twice as long as peduncle of uropod III	***G.zhouqiongi* sp. nov.**
–	Pereopod V–VII are strong and inner ramus uropod III less than twice as long as peduncle of uropod III	***G.tastiensis***

## Supplementary Material

XML Treatment for Gammarus

XML Treatment for Gammarus zhouqiongi
